# Reply to Brandt et al., “Antibodies to Nucleocapsid Are Not Diagnostic for Long COVID”

**DOI:** 10.1128/spectrum.00065-23

**Published:** 2023-02-14

**Authors:** Kamille Fogh, Peter Garred, Cecilie B. Hansen, Kasper K. Iversen

**Affiliations:** a Department of Cardiology, Copenhagen University Hospital, Herlev and Gentofte, Herlev, Denmark; b Department of Emergency Medicine, Copenhagen University Hospital, Herlev and Gentofte, Herlev, Denmark; c Department of Clinical Medicine, Copenhagen University Hospital, Faculty of Health and Medical Sciences, Copenhagen, Denmark; d Laboratory of Molecular Medicine, Department of Clinical Immunology, Rigshospitalet, Copenhagen, Denmark; Thomas Jefferson University

## REPLY

We appreciate the interest, queries, and comments from Brandt et al. on the *Microbiology Spectrum* article, “Self-reported long COVID and the association to SARS-CoV-2 antibodies in a Danish cohort up to 12 months after infection” (1). Brandt et al.’s concerns center on inclusion of PCR results, participation, and antibody persistence. To provide adequate clarification, we have addressed each query separately in the sections that follow.

The first question posed by Brandt et al. concerns the small number of respondents (less than 5%) and skewed gender distribution. We had a target population of 8,000 members of the social media group, but likely due to logistics, as blood samples could only be taken at 3 different hospitals in Denmark, our study population ended up with 350 participants, which is 5% of the target population. We did not anticipate that the entire target group would participate. In total, 926 social media users expressed an interest in participating, and 350 (38%) of them had a blood sample taken, with sampling bias as a limitation. As stated in the article’s limitations section, participation bias cannot be ruled out because members of the social media group were more often women than men.

The second question posed by Brandt et al. concerns the long-term persistence of nucleocapsid protein (N protein). According to Brandt et al., 30 to 40% of patients will not have persisting antibodies at the time of recruitment, and antibody decay is faster with mild COVID. In our study, we are looking at observations and measuring the qualitative outcome (seropositive or seronegative) rather than the quantitative outcome. The presence of SARS-CoV-2 N protein total Ig was determined using Roche electrochemiluminescence and a COBAS analyzer system, as mentioned in the article. This assay has been shown to be reliable, with a specificity of 98.1% compared to prepandemic samples and a sensitivity of 99.1% in patients with a confirmed PCR-positive test 14 days after symptom start (2). There was no significant difference in assay sensitivity by disease severity. Thus, the gray area of whether we oversee true-positive samples appears to be negligible. According to a published study, detectable N protein can be found up to 6 months after infection, with an increasing tendency due to improved affinity (3). The same system was used in an unpublished study of hospital employees from our group who were mostly infected but not hospitalized. In this study, N protein was found in 90% of previously infected participants after 12 months. It should be noted that N protein seropositivity and decline over time may vary between different studies, as other studies have shown around 80% seropositivity 9 months after virologically confirmed SARS-CoV-2 infection (4).

The third question posed by Brandt et al. concerns the inclusion of PCR test-positive patients in the characterization of long COVID. Brandt et al. mention that 90% of the participants reported a previous PCR test, and the inclusion of the PCR result could be used as a reliable factor in the characterization of long COVID. Unfortunately, the questionnaire design only allowed participants to report the most recent PCR test, which was not always related to the first reported infection with SARS-CoV-2. As a result, we chose not to include self-reported PCR test results.

One of the key points of our paper is that because PCR testing was not widely accessible at the beginning of the pandemic, participants who reported infection at the beginning of the period would not have been able to report a positive PCR result.
